# Seroprevalence and Associated Risk Factors of Peste des Petits Ruminants Virus in Small Ruminants of Punjab, Pakistan

**DOI:** 10.1155/tbed/7531764

**Published:** 2026-01-19

**Authors:** Mughees Aizaz Alvi, Hong-Bin Yan, Rai Bahadur Kharl, Aliza Ali, Muhammad Zeeshan, Wan-Zhong Jia, Shanhui Ren, Xuelian Meng, Xueliang Zhu, Muzafar Ghafoor, Muhammad Saqib, Li Li, Yongxi Dou

**Affiliations:** ^1^ State Key Laboratory for Animal Disease Control and Prevention, College of Veterinary Medicine, Lanzhou University, Gansu Province Research Center for Basic Disciplines of Pathogen Biology, Key Laboratory of Veterinary Parasitology of Gansu Province, Key Laboratory of Veterinary Etiological Biology and Key Laboratory of Ruminant Disease Prevention and Control (West), Ministry of Agricultural and Rural Affairs, National Para-reference Laboratory for Animal Echinococcosis, Lanzhou Veterinary Research Institute, Chinese Academy of Agricultural Sciences, Lanzhou, 730046, China, caas.cn; ^2^ Department of Clinical Medicine and Surgery, University of Agriculture, Faisalabad, Pakistan, uaf.edu.pk; ^3^ Department of Parasitology, University of Agriculture, Faisalabad, Pakistan, uaf.edu.pk; ^4^ Department of Pathobiology, Riphah College of Veterinary Sciences, Riphah International University, Lahore, Pakistan, riphah.edu.pk

**Keywords:** competitive ELISA, epidemiological modeling, goat, PPRV, sheep

## Abstract

Peste des petits ruminants (PPR) is a highly contagious viral disease prevalent in sheep and goats, and causes significant economic losses. The study was conducted in 2024 in Punjab Province, Pakistan, to estimate the seroprevalence of the PPR virus (PPRV) and to analyze animal‐level risk factors in unvaccinated small ruminants. Over a 12‐month period, multistage random sampling provided 722 serum samples of sheep and goats aged 6 months or older, collected across six districts. The anti‐PPRV antibodies were detected using competitive enzyme‐linked immunosorbent assay (cELISA), and species, age, sex, breed, parity, lactation status, pregnancy status, body condition score (BCS), and reproductive history were analyzed using univariable and multivariable logistic regression analyses. The overall seroprevalence rate was 79.77%, but significantly higher in goats (90.27%) than in sheep (68.75% and *p*  < 0.0001). The results demonstrated district wise disparity, with variation in seroprevalence between districts: 52.05% (Okara) and 100% (Nankana). An increased likelihood of seropositivity was found to be associated with male sex, some breeds (Makhi Cheeni and Beetal), certain species‐district interactions, and age‐sex interactions. These data confirm the high endemicity of PPRV in Punjab and justify the targeted vaccination and surveillance in high‐risk areas and among susceptible animal populations.

## 1. Introduction

Peste des petits ruminants (PPR) is a transmissible viral disease that has profound negative effects on the health of small ruminants and their productivity while causing severe economic damage in affected regions [1]. The causative agent, PPR virus (PPRV), a member of *Morbillivirus* genus within the Paramyxoviridae family, causes high fever along with signs related to pneumoenteritis, including mucopurulent nasal discharge, stomatitis, diarrhea, and pneumonia, which often lead to high death rates [2, 3]. A diverse set of factors including, host immunity, age, management practices and secondary infections, determine mortality rates, which typically range between 50% and 90% [4, 5].

Studies on virus classification have revealed four distinct lineages designated I through IV, with Lineage IV representing the most prevalent strain in Asia [6–8]. The disease emerged in Côte d’Ivoire in 1942 [9] and is now endemic in more than 70 countries across Asia and Africa [10]. The World Organisation for Animal Health (WOAH) declared PPR a notifiable disease, as it poses major risks to food security, livelihoods, and rural economies [5, 9]. PPR has become a target for global eradication by 2030 because of its socioeconomic impact, transboundary nature, and effects on food security [11]. The global goal to eradicate PPR by 2030 encounters major obstacles because vaccination programs remain irregular and disease monitoring lacks depth, diagnostic tools are limited, and animal transport is poorly managed [12–14].

The rural economy of Pakistan relies heavily on small ruminants, but PPR remains endemic throughout the country, particularly in regions where domestic livestock species live in close proximity and share feeding and water sources, thereby facilitating opportunities of viral transmission [15]. Seroconversion in cattle and buffaloes implies either undetected subclinical infections or contact of these animals with the virus [16, 17]. Sheep and goats represent the largest animal population in the agriculturally productive and densely populated Punjab province of Pakistan. Research findings from Punjab province showed antibody levels against PPRV in sheep and goats ranged from 30% to over 70%, while contrasting results were attributed to poor biosecurity practices and irregular vaccination programs [5, 18]. Quantifying antibody prevalence enables more accurate predictions about disease impact and supports the development of effective vaccination strategies and successful control programs.

Detection of previous PPRV exposure depends on measuring PPR antibodies which are present regardless of infection or vaccination history [19]. In Pakistan, routine vaccination against PPRV is practiced in the province of Punjab. However, reported coverage was neither uniform nor what can be considered satisfactory with district‐level surveys showing that only a little over one‐third of all small ruminants are vaccinated on a continual basis. Much of the failure to eliminate PPRV can be attributed to suboptimal levels of vaccination coverage, particularly in the rural, underserved areas, which have permitted endemic transmission even during the mass immunization campaigns.

Most of the studies that have identified seroprevalence of PPR in the Punjab province of Pakistan are limited in geographical scope and have not undertaken a thorough risk factor analysis, that is, the use of multivariate modeling to explain the effects of interaction. These methodological shortcomings limit the ability to outline vulnerable subpopulations and geographic targets for intervention. Therefore, the current study aimed to generate a district wide sero‐epidemiological profile of PPRV in unvaccinated ovine and caprine flocks in Punjab using a risk‐based analytical methodology. The results are to be used to provide evidence that can guide the development of more effective surveillance, vaccination, and control programs and contribute to the global goal of eliminating PPR by 2030.

## 2. Materials and Methods

### 2.1. Study Area and Study Design

**Figure 1:**
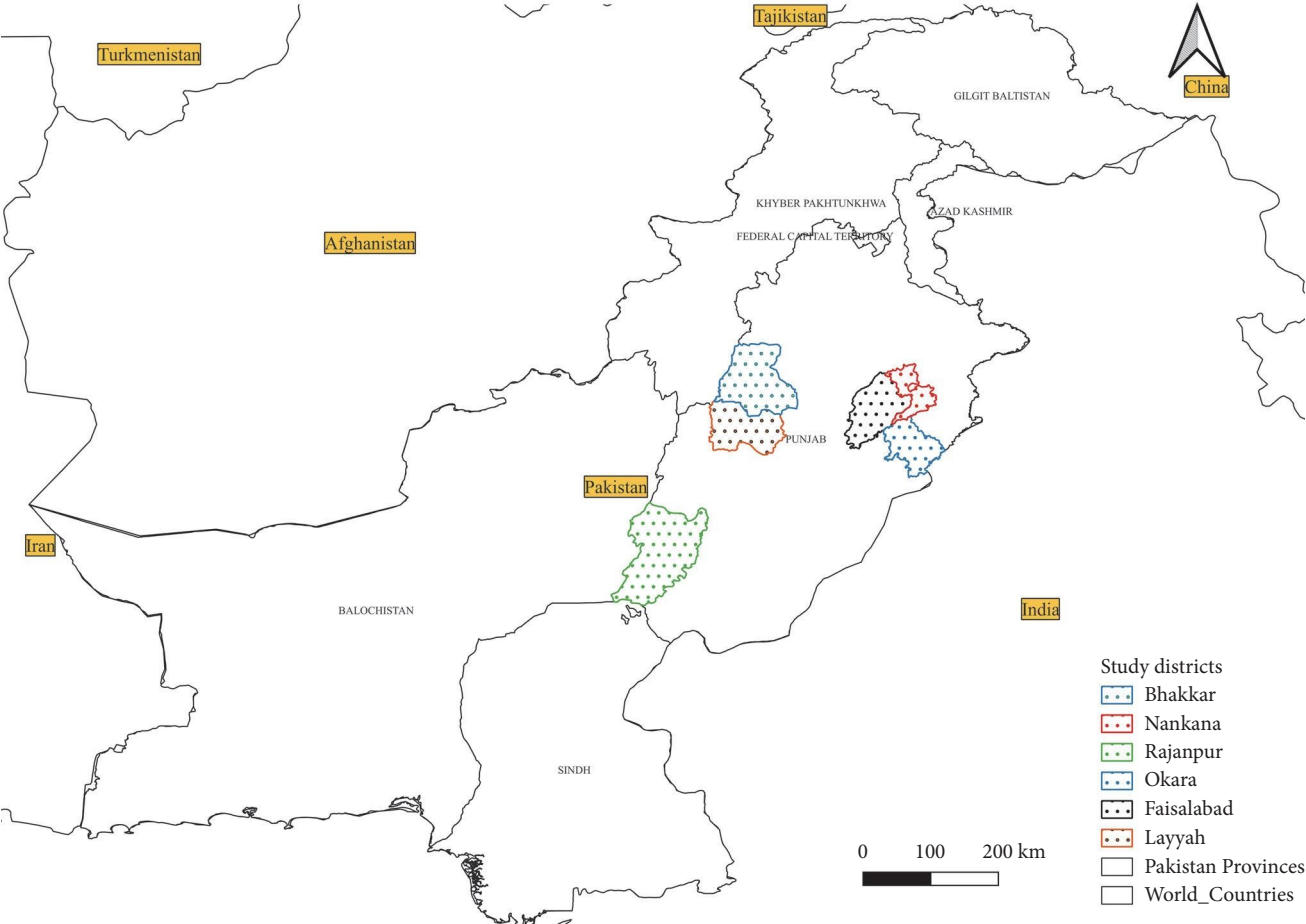
Map of Pakistan indicating the study districts.

The Punjab Province of Pakistan was selected for sampling since it is the most populous and agriculturally productive region of Pakistan that harbors the largest population of small ruminants (overall ~89.4 million goat heads and 31 million sheep heads in Pakistan) in the country. Punjab is also an animal movement and interdistrict trade hotspot, and therefore is highly relevant in respect of the transboundary diseases study. The Punjab Province of Pakistan has 36 districts with a subtropical climate and is characterized by significant seasonal differences, and this feature directly impacts the livestock health and disease conditions. A multistage random sampling process has been used to obtain data. In the first phase, the six districts (Okara, Faisalabad, Bhakkar, Rajanpur, Layyah, and Nankana) were randomly selected based on a 36 pool of districts in the province with an equal likelihood of selection (Figure 1).

A cross‐sectional study was conducted between January and December 2024 on unvaccinated sheep and goats older than 6 months to ensure that the detected antibodies were due to natural exposure to PPRV rather than vaccine‐induced immunity or maternal‐ transferred antibodies. In order to have a representative sample in the Punjab, three typical systems of small ruminant production were included, such as sedentary, nomadic, and mixed, using a multistage random sampling methodology based on the livestock census and district veterinary records.

 Stage 1‐Selection of districts: Punjab was stratified by production systems, and two districts were chosen randomly in each system: sedentary (Okara and Faisalabad), nomadic (Bhakkar and Rajanpur), and mixed (Layyah and Nankana).
 Stage 2‐Selection of villages: Four to six villages were randomly selected within each district using the official livestock office record to select the villages with a random number generator.
 Stage 3‐Selection of flocks: Flocks of sheep and goats were counted in each of the selected villages with the help of the local veterinary service. From these enumerations, two to four flocks were randomly selected.
 Stage 4‐Selection of individual animals: In each of the selected flocks, individual animals aged above 6 months and with no record of previous PPR vaccination were randomly selected to achieve a proportional sample size representative of the district.

Stage 1‐Selection of districts: Punjab was stratified by production systems, and two districts were chosen randomly in each system: sedentary (Okara and Faisalabad), nomadic (Bhakkar and Rajanpur), and mixed (Layyah and Nankana).

The study sample was obtained using a four‐stage design, which helped minimize selection bias while accounting for the geographic distribution and production systems of small ruminants in Punjab.

In earlier research in Punjab, it has been recorded that the PPRV seroprevalence rates range between 30% to 70%, leading us to assume an expected prevalence of 50%. Using 5% margin of error and a 95% confidence interval, the minimum sample size required to obtain a precise and reliable estimate was 384 animals. To ensure statistical power greater than 80% and to account for the multistage sampling scheme, the sample size was doubled, yielding a final estimate of approximately 720 small ruminants. A total of 722 animals were sampled, which was sufficient to achieve the desired level of precision statistical power. Vaccination status was verified through triangulation of farmer interviews and local veterinary office records. Only animals that had not been previously vaccinated were enrolled in the study.

### 2.2. Data Collection

The structured questionnaire (Supporting information S1) was given to flock owners during the sampling event. The questions were applied to each flock in the multistage random sampling plan. Data were recorded on standardized paper forms and then entered into Microsoft Excel. Laboratory technicians extracted demographic information from animals, including owner demographics, animal characteristics (species, breed, sex, age, body condition, and district), and reproductive data for females (parity, lactation, pregnancy status, and reproductive disorders). The body condition score (BCS) of each animal was also measured during sampling on a standard 1–5 scale, where 1 indicated emaciation, and 5 indicated obesity. The assessment relied on visual inspection and palpation of the lumbar vertebrae and the pelvis bones. The scoring is commonly used in small ruminant epidemiology literature (Body Condition Scoring of Sheep and Goats. Technical Bulletin No. 8. Ethiopia Sheep and Goat Productivity Improvement Program (ESGPIP) (http://40.65.112.141/AIGR/tb/TB%208%20Body%20condition%20scoring.pdf).

### 2.3. Sample Collection

In total, 722 animals were sampled, including 370 goats and 352 sheep. Each animal was aseptically punctured in the jugular vein to collect five milliliters of whole blood in a sterile plain (clot activator) vacutainer tube without anticoagulant. The tubes were left standing in an insulated icebox with frozen gel packs (4°C) for 1–2 h to allow clotting. Serum was then separated in the laboratory on the collection day by centrifugation at 3000 rpm for a duration of 10 min. The supernatant serum was transferred to sterile, labelled cryovials, immediately stored in the freezer, and then transported under strict cold chain conditions in insulated containers with dry ice to the Laboratory of Veterinary Preventive Medicine and Public Health, University of Agriculture, Faisalabad, Pakistan.

### 2.4. Serological Testing

The detection of PPRV antibodies in sera was carried through a commercially available competitive enzyme‐linked immunosorbent assay (cELISA) kit obtained from Lanzhou Veterinary Research Institute in China under Patent Number ZL201210278970.9. The kit is documented to have a sensitivity of 84% and a specificity of 97.7% as established in a comparative analysis done internally at LVRI (*n* = 176 sera) with the commercial ID‐Screen PPR Competition ELISA (ID‐Vet), data cited in the literature [5].

The procedure followed the instructions provided by the manufacturer at the same step as [20].

The percentage inhibition (PI) was calculated using the formula:

PI%=1−OD of sample/OD of negative control×100.

Interpretation criteria were as follows:

• PI ≥ 50%: positive
• PI < 50%: negative

### 2.5. Data Management and Statistical Analysis

The collected data were entered into Microsoft Excel prior to analysis using SPSS Version 20. Prevalence was calculated using descriptive statistical methods. The research team performed both univariable and multivariable logistic regression analyses to assess the association between potential risk factors and PPR serological results, with *p*  < 0.05 statistically significant [21]. Associations were assessed through odds ratios (OR) alongside 95% confidence intervals (CIs). Male animals were not included in analyzing the risk factors that are female‐specific, like parity, lactation status, pregnancy status, and history of reproductive disorders. This choice of methodology improved the internal validity of the study.

The fitted multivariable logistic regression model used PPRV serologic status, as determined by cELISA (positive versus negative), as the outcome variable, and predicted probabilities of ELISA positivity were extracted from the model, with linear predictors translated into probabilities using the inverse logit transformation. The resulting values were summarized and graphically presented to illustrate risk distribution across categories such as species, sex, district, breed, and age.

PPRV serological status, as determined by cELISA, was treated as a binary outcome variable. Explanatory factors included species (sheep or goat), sex (male or female), age (categorized), breed (categorical), sampling districts, BCS, and reproductive factors such as parity, pregnancy status, lactation status, and reproductive history. In the chi‐square analysis, categorical explanatory variables were cross‐tabulated against serologic status to test for statistical independence, with the reference category specified as the one with the lowest observed prevalence.

### 2.6. Ethics Approval and Consent to Participate

This study was carried out in accordance with the national and institutional animal care and use regulations. All animal procedures were conducted under aseptic conditions and under the supervision of licensed veterinarians. Blood sampling was performed by inserting the needle into the jugular vein only to the point of minimal blood requirement (5 mL) to preserve animal welfare. No anesthetics or harmful procedures were used, and animals were restrained using the minimum force possible to reduce discomfort and stress.

All livestock owners provided verbal informed consent to participate in the research before sampling. Interviews were conducted clarifying the purpose, procedures, and how the data will be used. The research was conducted as part of a standard veterinary field survey of PPR and in accordance with the University of Agriculture, Faisalabad, Pakistan. According to this policy, all nonexperimental standard diagnostic sampling is exempt from the formal review process. However, all contacts with animals and interactions with farmers were conducted following internationally accepted principles of animal welfare and research ethics.

## 3. Results

### 3.1. Overall Seroprevalence

The cELISA test for detecting anti‐PPRV antibodies was conducted on 722 sheep and goat serum samples. Of these, 576 tested positive, resulting in an overall prevalence of 79.77%. Detailed univariate analysis of anti‐PPRV antibodies and associated risk factors is presented in Table 1, while the analysis of risk factors specific to females is mentioned in Table 2. For each categorical variable, the subgroup with the lowest observed seroprevalence was defined as the reference category, allowing the OR to indicate relative prevalence compared to the least affected subgroup.

**Table 1:** Univariate analysis of risk factors associated with PPRV seroprevalence in sheep and goats in Punjab, Pakistan.

Category	Variable	Positive/tested	Prevalence (95% CI)	Odds ratio (95% CI)	Chi‐square value (*p*‐value)
District	Nankana	33/33	100 (89.70–100)	1.92 (1.04–3.56)	*χ* ^2^ = 49.03 (*p* < 0.0001)
	Bhakkar	75/85	88.23 (79.71–93.49)	1.70 (1.03–2.79)	
	Faisalabad	283/343	82.50 (78.14–86.16)	1.59 (1.04–2.41)	
	Rajanpur	30/37	81.08 (65.69–90.52)	1.56 (0.84–2.89)	
	Layyah	117/151	77.48 (70.18–83.41)	1.49 (0.94–2.35)	
	Okara	38/73	52.05 (40.78–63.12)	Reference	

Species	Goats	334/370	90.27 (86.82–92.89)	1.31 (1.05–1.64)	*χ* ^2^ = 50.46 (*p* < 0.0001)
	Sheep	242/352	68.75 (63.73–73.37)	Reference	

Sex	Male	217/253	85.77 (80.93–89.54)	1.12 (0.89–1.41)	*χ* ^2^ = 8.11 (*p* = 0.0044)
	Female	359/469	76.54 (72.51–80.16)	Reference	

Age	8–10 years	12/14	85.71 (60.05–95.99)	1.17 (0.54–2.58)	*χ* ^2^ = 10.91 (*p* = 0.0276)
	6–8 years	45/53	84.90 (72.95–92.15)	1.16 (0.74–1.83)	
	6 months to 2 years	304/364	83.51 (79.36–86.98)	1.14 (0.88–1.49)	
	4–6 years	72/95	75.78 (66.28–83.29)	1.04 (0.71–1.51)	
	2–4 years	143/196	72.95 (66.34–78.69)	Reference	

Breed	Makhi Cheeni	95/95	100 (96.11–100)	4.89 (2.28–10.50)	*χ* ^2^ = 200.89 (*p* < 0.0001)
	Beetal	129/132	97.72 (93.54–99.23)	4.78 (2.26–10.12)	
	DDP	24/26	92.30 (75.86–97.87)	4.51 (1.84–11.08)	
	Rajanpuri	29/32	90.62 (75.79–96.76)	4.43 (1.86–10.55)	
	Teddy	23/26	88.46 (71.02–96.00)	4.32 (1.76–10.65)	
	Mundri	51/63	80.95 (69.59–88.75)	3.96 (1.78–8.81)	
	Nachy	26/34	76.47 (60.00–87.56)	3.74 (1.56–8.94)	
	Thalli	88/115	76.52 (67.99–83.33)	3.72 (1.75–8.02)	
	Kajli	94/129	72.86 (64.62–79.80)	3.56 (1.67–7.60)	
	Sindhi	8/26	30.76 (16.50–49.99)	1.50 (0.52–4.32)	
	Lohi	9/44	20.45 (11.15–34.50)	Reference	

Body condition score (BCS)	3	396/474	83.54 (79.93–86.61)	8.35 (2.01–34.78)	*χ* ^2^ = 75.67 (*p* < 0.0001)
	4	13/16	81.25 (56.99–93.41)	8.13 (1.65–39.92)	
	2	164/207	79.22 (73.20–84.20)	7.92 (1.89–33.27)	
	1	1/5	20 (3.62–62.45)	2 (0.18–22.50)	
	5	2/20	10 (2.79–30.10)	Reference	

*Note*: The odds ratio (OR) measures the ratio of odds that each PPRV seropositivity category has compared to the reference category. Chi‐square values represent the comparison of ELISA seropositivity (positive vs. negative) across the respective categories of each variable. Significant *χ* ^2^ *p*‐values may occur even if individual category ORs are not significant, because the test evaluates the collective differences among all groups within the variable.

**Table 2:** Univariate analysis of female‐related risk factors associated with PPRV seroprevalence in sheep and goats in Punjab, Pakistan.

Category	Variable	Positive/tested	Prevalence (95% CI)	Odds ratio (95% CI)	Chi‐square value (*p*‐value)
Parity	9th	3/3	100 (43.85–100)	1.49 (0.33–6.71)	*χ* ^2^ = 8.25 (*p* = 0.5092)
	7th	7/8	87.50 (52.91–97.76)	1.30 (0.46–3.70)	
	6th	13/15	86.66 (62.12–96.27)	1.29 (0.57–2.91)	
	8th	5/6	83.33 (43.65–96.99)	1.24 (0.38–4.07)	
	5th	26/32	81.25 (64.69–91.11)	1.21 (0.65–2.27)	
	Zero	121/152	79.60 (72.51–85.25)	1.19 (0.77–1.83)	
	3rd	41/56	73.21 (60.40–83.04)	1.09 (0.64–1.87)	
	4th	37/48	77.08 (63.46–86.69)	1.15 (0.66–2.01)	
	1st	57/76	75.00 (64.22–83.37)	1.12 (0.68–1.84)	
	2nd	49/73	67.12 (55.73–76.70)	Reference	

Lactation status	Nonlactating	221/279	79.21 (74.07–83.56)	1.09 (0.82–1.44)	*χ* ^2^ = 12.03 (*p* = 0.0073)
	Lactating	138/190	72.63 (65.89–78.47)	Reference	

Pregnancy status	Nonpregnant	253/320	79.06 (74.27–83.16)	1.11 (0.82–1.50)	*χ* ^2^ = 3.05 (*p* = 0.2171)
	Pregnant	106/149	71.14 (63.41–77.81)	Reference	

Reproductive disorders	Stillbirth	12/13	92.30 (66.69–98.63)	1.45 (0.44–4.81)	*χ* ^2^ = 11.01 (*p* = 0.3569)
	Nil	303/393	77.09 (72.70–80.98)	1.21 (0.48–3.08)	
	Abortion	30/41	73.17 (58.07–84.30)	1.15 (0.41–3.23)	
	Premature delivery	7/10	70.00 (39.68–89.22)	1.10 (0.30–4.09)	
	Repeat breeders	7/12	58.33 (35.38–84.84)	Reference	

*Note*: The odds ratio (OR) measures the ratio of odds that each PPRV seropositivity category has compared to the reference category. Chi‐square values represent the comparison of ELISA seropositivity (positive vs. negative) across the respective categories of each variable. Significant *χ* ^2^ p‐values may occur even if individual category ORs are not significant, because the test evaluates the collective differences among all groups within the variable.

The OR and their respective 95% CI were calculated for each for each diagnostic category compared with the reference category in the present study. The chi‐square value and the *p*‐value indicate the overall statistical association between the variable and PPRV seropositivity across all categories.

The real estimate of seroprevalence calculated by the Rogan and Gladen formula, with the sensitivity (84%) and the specificity (97.7%) of the serological test, was 94.8% among the small ruminant populations in Punjab. The corrected prevalence in goats was 100% (apparent 90.27%; adjusted estimate over 100% truncated to 100%), while in sheep it was 81.3% compared with an apparent seroprevalence of 68.75%. Statistical corrections suggested that the higher apparent seroprevalences observed in this study are a valid reflection of a widespread exposure to PPRV among small ruminants in Punjab.

### 3.2. District Wise Seroprevalence

**Figure 2:**
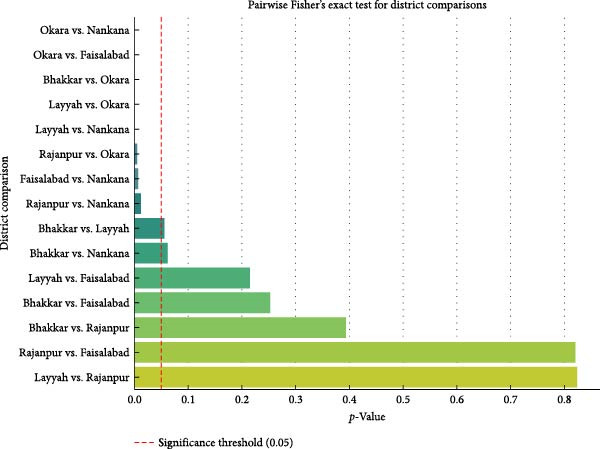
Pairwise Fisher’s exact test results for district‐wise comparisons of ELISA positivity.

District wise testing for PPRV antibodies revealed varying detection rates across the surveyed districts in Punjab, Pakistan. A total of 33 animals were tested at Nankana, resulting in complete (100%) seropositivity. The 85 animals tested from Bhakkar district recorded 75 positive cases, indicating an 88.23% seroprevalence. A total of 343 animals underwent testing in Faisalabad, where 82.50% (*n* = 283) tested positive for anti‐PPRV antibodies. Results from Layyah also documented a high prevalence of PPRV antibodies at 77.48%, where 117 from 151 examined animals showed positive results. The Okara district had the lowest percentage of seropositive cases at 52.05%, since 38 animals out of 73 tested positive. Thirty of 37 tested animals showed a positive result in Rajanpur district, having a prevalence of 81.08%. A strong relationship between district and seroprevalence levels (*χ* ^2^ = 49.03, *p*  < 0.0001) was found. PPRV antibody prevalence rates vary extensively throughout the different districts of Pakistan, according to this data. For districts, the pairwise post hoc Fisher’s exact test recognized major differences in seroprevalence patterns that emerged between multiple pairs of districts. The pairwise post hoc Fisher’s exact test for district demonstrated highly significant differences (*p*  < 0.01) between district pairs including Okara and Faisalabad (*p*  < 0.0001), Okara and Nankana (*p*  < 0.0001), Layyah and Okara (*p* = 0.0002), Layyah and Nankana (*p* = 0.0009), and Rajanpur versus Okara (*p* = 0.0036) (Figure 2). The study findings reveal specific epidemiological profiles and risk elements operating between these districts. A moderate level of statistical difference (*p*  < 0.05) was detected through analysis of serum tests between Faisalabad and Nankana (*p* = 0.0046), and Rajanpur and Nankana (*p* = 0.0122). The comparison of Bhakkar to Layyah districts produced (*p* = 0.0547) and Bhakkar to Nankana produced (*p* = 0.0600). The seroprevalence rates of these district pairs showed no statistical difference according to the analysis results (*p*  > 0.05).

Figure 2 shows the analysis of *p*‐values from Fisher’s exact tests through a bar plot demonstrating ELISA positivity differences between districts. This figure includes a red dashed line, which serves as the *p* = 0.05 threshold for significance. Any districts with pairwise comparisons that fall below this red dashed line show significant differences in seroprevalence rates for the respective territory. Bar color intensity reflects the degree of statistical significance, with darker colors indicating lower *p*‐values (stronger evidence of difference between districts).

### 3.3. Species‐Specific Seroprevalence

Results from the seroprevalence analysis demonstrated distinct differences between goat and sheep populations. The comparison of seroprevalence between sheep and goats revealed a highly significant difference (*χ* ^2^ = 50.46, *p*  < 0.0001), with goats showing higher antibody prevalence (90.27%; 334/370) compared to sheep (68.75%; 242/352). This indicates distinct seroprevalence trends across species, likely reflecting differences in susceptibility, husbandry, and exposure patterns.

### 3.4. Age‐Related Seroprevalence

**Figure 3:**
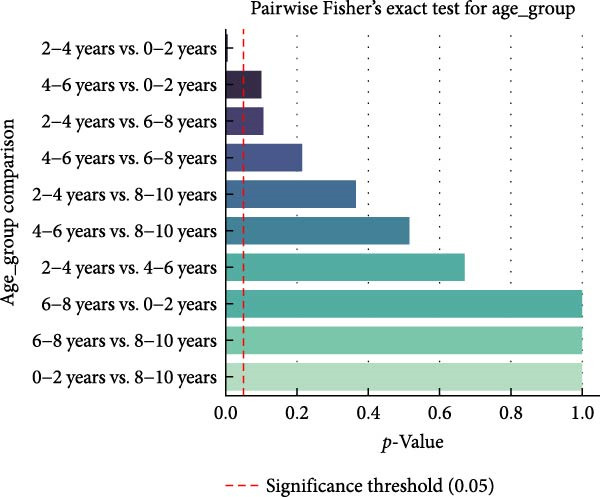
Pairwise Fisher’s exact test results for age group comparisons in ELISA positivity. The red dashed line shows the *p* = 0.05 mark of statistical significance.

The study showed 83.51% (304 positive of 364 tested) animals aged 6 months to 2 years were positive for antibodies. The seroprevalence results of animals aged between 2 and 4 years were slightly lower at 72.95% as 143 out of 196 animals tested positive for antibodies. Animals between the ages of 4–6 years displayed a seroprevalence rate of 75.78% (72 positive from 95 tested animals), and older flock members (6–8 years group) displayed an elevated rate of 84.90% (45 positive from 53 tested). The oldest animal cohort of 8–10 years showed the highest seropositive rate at 85.71% based on 12 out of 14 positive results, which implies growing antibody levels. Serological data analysis revealed that the age group proved statistically significant for seropositivity results (*χ* ^2^ = 10.91, *p* = 0.0276). Numerous statistically important contrasting patterns emerged from pairwise comparisons. Younger animals tested significantly different from animals between 2 and 4 years old. The oldest age groups (8–10 years) revealed significant differences in antibody detection compared to younger age categories probably due to accumulating exposure and prolonged immunity. The *p*‐values of pairwise Fisher’s Exact Tests analyzing ELISA positivity between different age groups appear in Figure 3. Apart from the significance threshold at *p* = 0.05, the red dashed line serves as a guide. Significant statistical differences emerged from pairwise comparisons between animals in the 0–2 year age category and those in the 2–4 and 4–6 year groups, indicating dissimilar seroprevalence profiles between younger and older groups.

### 3.5. Sex‐Related Seroprevalence

Analysis showed that males showed higher PPRV exposure results than analysis of females. The study revealed that 76.54% (359 out of 469) of tested female animals had PPRV antibodies, while PPRV seropositivity among male animals reached 85.77% (217 out of 253). The majority of animals positive for PPRV showed serologic responses based on their sex identity (*χ* ^2^ = 8.11, *p* = 0.0044), with males demonstrating elevated PPRV seroprevalence rates.

### 3.6. Breed‐Specific Seroprevalence

Breed‐specific seroprevalence revealed substantial variability. Beetal breed suffered markedly high levels of antibody detection because 97.72% of its 132‐member population (129 animals) tested positive. We found even higher prevalence levels in Makhi Cheeni breed. The Rajanpuri breed revealed a seroprevalence rate of 90.62% (29 positive animals out of 32 tested), followed by the DDP breed with 92.30% seropositivity (24 positive out of 26 tested animals), then by the Teddy breed with 88.46% seroprevalence (23 positive out of 26 animals). Mundri, Nachy, and Thalli breed populations reported prevalence results at 80.95%, 76.47%, and 76.52%, respectively. The Kajli breed demonstrated 72.86% seropositivity in which 94 positive animals emerged from 129 total tests. Only 20.45% of Lohi sheep were positive from a total of 44 animals evaluated. Research results demonstrated that Sindhi breed contained relatively few infections since eight out of 26 tested animals tested positive with a 30.76% prevalence rate. Statistics showed Breed as the strongest single factor behind seroprevalence (*χ* ^2^ = 200.89, *p*  < 0.0001) thus indicating variation between breeds pertaining to exposure or inherent susceptibility patterns. A comparison of different breeds showed many statistically significant pairwise differences between groups. The seropositivity rate was higher among Makhi Cheeni and Beetal as well as DDP and Rajhanpuri breeds when compared to Lohi and Sindhi breeds.

### 3.7. BCS

There was a strong correlation between disease seropositive and BCS. The greatest seroprevalence was among animals whose BCS was three (83.54%), then four (81.25%), and two (79.22%). On the contrary, animals in the poor body condition (BCS = 1) and those in the excellent body condition (BCS = 5) had significantly lower prevalence rates of 20% and 10%, respectively.

### 3.8. Parity and Lactation Status

The serological test results indicated 121 out of 152 animals without parity generated positive results (79.60% seropositivity). Tests revealed that animals with parity levels one, two, and three were seropositive in 57 out of 76 animals (75.0%), 49 out of 73 animals (67.12%), and 41 out of 56 animals (73.21%), respectively. Among the studied animals those with parity levels four through eight demonstrated the higher seroprevalence rates of 77.08% (37 out of 48 animals), 81.25% (26 out of 32 animals), 86.66% (13 out of 15 animals), 87.50% (seven out of eight animals), and 83.33% (five out of six animals), respectively. All tests performed on animals with parity nine revealed full seropositivity, resulting in 100% positive results, indicating either antibody buildup or a higher rate of exposure. The analysis showed parity made no difference in seropositivity measures (*χ* ^2^ = 8.25, *p* = 0.5092) (Table 2). The presence of antibodies might not depend strongly on parity status alone.

The analysis of lactation status showed distinct results between lactating and nonlactating animals. The serological examination of 190 lactating females confirmed that 138 animals carried the virus for a total prevalence rate of 72.63%. Out of 279 nonlactating female animals tested, 79.21% revealed positive results for anti‐PPRV antibodies. Lactation status showed a meaningful link (*χ* ^2^ = 12.03, *p* = 0.0073) because lactation states affected how seroprevalence patterns unfolded. The marked variation between lactating and nonlactating sheep and goats could be linked to physiological differences (e.g., immunosuppression during lactation) or management‐related factors.

### 3.9. Pregnancy Status and Reproductive Disorders

Different pregnancy conditions resulted in only minor differences in positive anti‐PPR antibodies cases. The analysis revealed that nonpregnant animals tested positive in 79.06% (253 of 320) were exposed to PPRV. Out of 149 pregnant animals examined, 106 tested positive for anti‐PPRV antibodies, yielding a 71.14% prevalence rate. The research results indicated pregnancy status was unrelated to seroprevalence levels (*χ* ^2^ = 3.05, *p* = 0.2171) (Table 2).

The serological prevalence of reproductive conditions displayed different measurement results. The seropositivity in the animals that had an abortion history was 73.17%. Animals that had no history related to reproductive disorders showed seroprvalence of 77.09% (303 of 393 animals tested). Results showed that animals showing premature delivery had a serology detection rate of 70.00%. Of the tested ‘repeat breeder’ animals, a 58.33% (7/12) prevalence was found. The highest seropositivity rate (92.30%) was found in the animals that had a history of stillbirth. The exposure history of certain animals to PPRV seems to become linked with their reproductive problems since these animals tested positive through ELISA methods.

### 3.10. Multivariable Logistic Regression

The results from the logistic regression analysis show these factors that affect the chance of testing positive for PPRV.

 District: PPRV detection rates show significant differences across locations according to district‐specific analysis (*p*  < 0.001).
 Species: The analysis shows one specific species, either goats or sheep, demonstrating a highly significant (*p*  < 0.001) negative statistical relationship (−1.6453) to PPRV positivity status.
 Sex: The results show females or males exhibit a positive likelihood of being positive while maintaining a significant impact at *p* = 0.0004.
 Breed: not statistically significant (*p* = 0.14).
 Age: This model shows no significant relationship between age and PPRV infection status (*p* = 0.43). Statistical analysis reveals the model fit with a pseudo *R* ^2^ value of 0.131 and an overall *p*‐value (LLR p) of approximtly equal 5.8 × 10^−19^.

District: PPRV detection rates show significant differences across locations according to district‐specific analysis (*p*  < 0.001).

### 3.11. Correlation and Predictive Modeling

**Figure 4:**
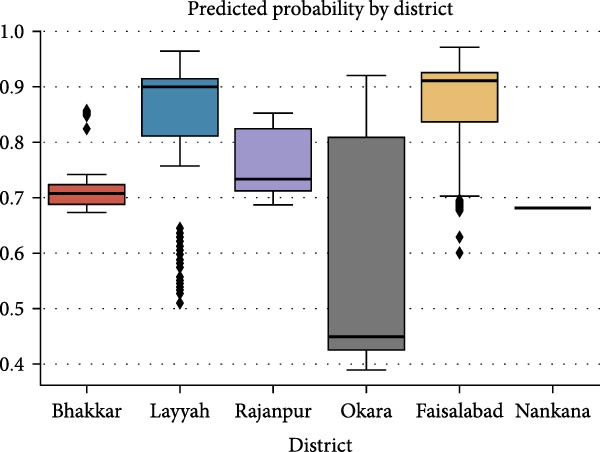
Predicted probability of ELISA positivity by district. Predicted probabilities are mentioned along *y*‐axis.

A logistic regression model was used to produce this boxplot (Figure 4) which analyzes predicted probabilities for ELISA positivity across various districts. During our analysis, Faisalabad and Layyah presented greater median predicted probabilities accompanied by reduced interquartile range variability, supporting their consistently higher risk composition. Analysis of Okara’s district data revealed both a larger type of distribution and lower median risk which demonstrates substantial unpredictability in risk levels across the region.

**Figure 5:**
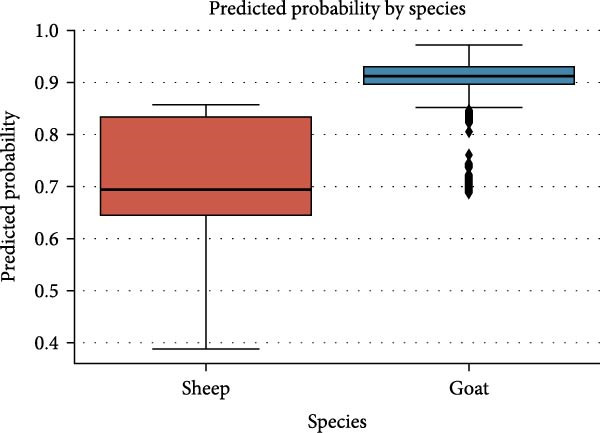
Predicted Probability of ELISA positivity by species. Predicted probabilities are mentioned along *y*‐axis.

Figure 5 shows predicted ELISA positivity probabilities among sheep along with goats. A logistic regression model showed goats carried higher predicted probabilities of ELISA positivity with less spread than sheep, which suggests goats face greater and more consistent exposure to the virus.

**Figure 6:**
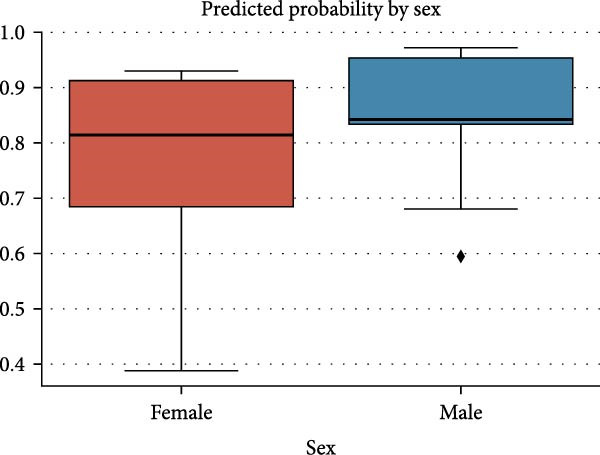
Predicted probability of ELISA positivity by sex. Predicted probabilities are mentioned along *y*‐axis.

In Figure 6, the predicted ELISA positivity outcomes per sex group are shown. The median predicted probabilities among males exceeded those of females, while their prediction range remained tighter. The current model indicates more variation in susceptibility to infections within the female population compared to males.

**Figure 7:**
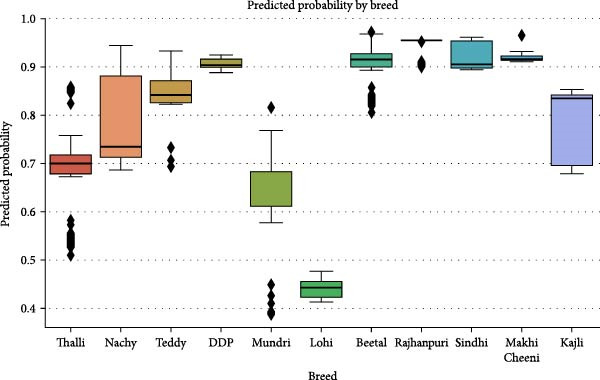
Predicted probability of ELISA positivity by breed.

In Figure 7, the graphical display shows predicted probabilities for ELISA test outcome variations across multiple types of livestock species. Beetal, Rajanpuri, and Kajli breeds demonstrate steady high predicted ELISA‐positive outcomes, yet Lohi and Mundri breeds tend toward lower median probability rates. The varying ranges of probability values observed within Nachy and DDP breeds demonstrate how infection risk differs among individual animals within the same breed group. This demonstrates the breed’s importance as a predictor of seropositivity.

**Figure 8:**
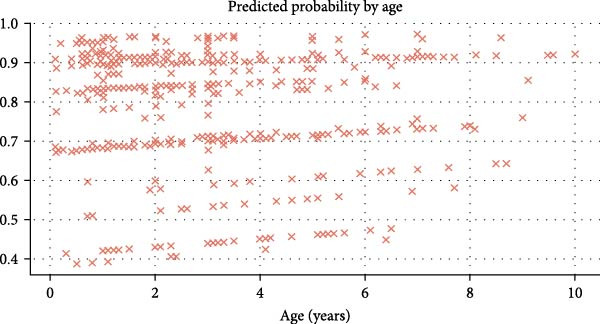
Predicted probability of ELISA positivity by age. Predicted probabilities are mentioned along *y*‐axis.

The scatter plot (Figure 8) represents the age correlation (in years) against the predicted probability of ELISA positivity. A majority of animals display predictable high probability values across every age group, although variations appear more noticeable in both young and older populations. The trend analysis reveals no definitive linear progression, indicating that age effects on seropositivity risks follow a complex pattern.

### 3.12. Interaction Effects Between Variables

By using interaction terms within multivariate logistic regression, we obtained deeper details regarding the factors linked to PPRV seropositivity. Laboratory results confirmed that sex stands as an independent predictor factor for PPRV positivity (*p* = 0.0092). The statistical influence of breed became meaningful when analyzed with other factors (*p* = 0.0272), indicating its effects appear in particular combinations of variables. The probability of PPRV infection shows substantial variation based on species composition and geographic district, according to this highly statistically significant interaction (*p* = 0.0015). The analysis showed that age‐related PPRV susceptibility levels differ between males and females (*p* = 0.0227).

When including interactions in the analysis, both variables lost prediction power as district variable became statistically insignificant (*p* = 0.7268) and species alone dropped to (*p* = 0.1992). This reveals the crucial importance of examining the combined effects of variables. Statistical significance was absent when analyzing the combined effect of animal sex with breed type. After adding interaction terms the model gained greater predictive ability as the pseudo *R* ^2^ value rose from 0.131 to 0.150. A robust statistical fit exists between the data and the overall model, which is shown by its extreme significance level (*p* ≈ 5.35 × 10^−20^).

**Figure 9:**
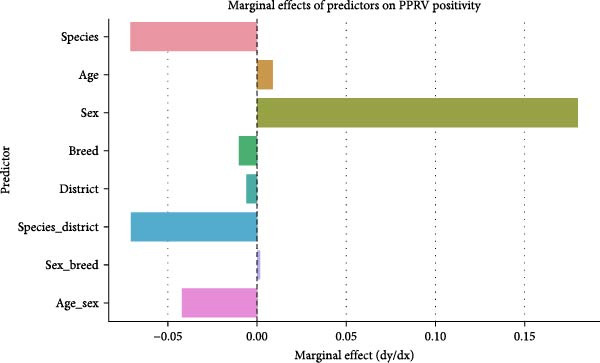
Marginal effects of predictors on PPRV positivity.

Figure 9 presents the standalone impact each predictor, together with their relationship combinations, has on PPRV positivity likelihood. Sex emerged as the strongest variable positively affecting PPRV seropositivity predictions among all examined factors. Positive effects from both species–district interaction and age–sex interaction, together with negative effects from species association, supported their importance for predicting infection risk. Additional variables, including age, breed, and district, showed inferior impact compared to other predictive elements.

A one‐unit change in each predictor leads to adjustments in the estimate of PPRV seropositivity probability according to the marginal effects plot after accounting for constant control variables. Sex exhibits the highest positive marginal effect, thus demonstrating significant elevations in PPRV positivity outcomes between male and female animals. The species × district and age × sex interaction terms produced significant negative effects that reduced infection probability across chosen subsets of the population. The observed impact from breed was significant despite being smaller compared to other variables, which played a role in determining infection risk levels.

A correlation heatmap was produced to analyze relationships between animal‐level variables with both numerical data and encoded categories. The ELISA result demonstrated a mild relationship with species‐type and breed‐species categories and geographical district data to support past findings about PPRV antibody identification patterns. The ELISA testing results showed minimal predictive value when correlated with age, parity, body condition, and reproductive status variables. Multiple regression modelling techniques, such as logistic regression, benefit from variables that lack significant covariance relations. The correlation results indicate geographic locations, together with genetic factors (district, breed, and species), exert more influence on PPRV serostatus than individual physiological traits (e.g., age and body condition).

**Figure 10:**
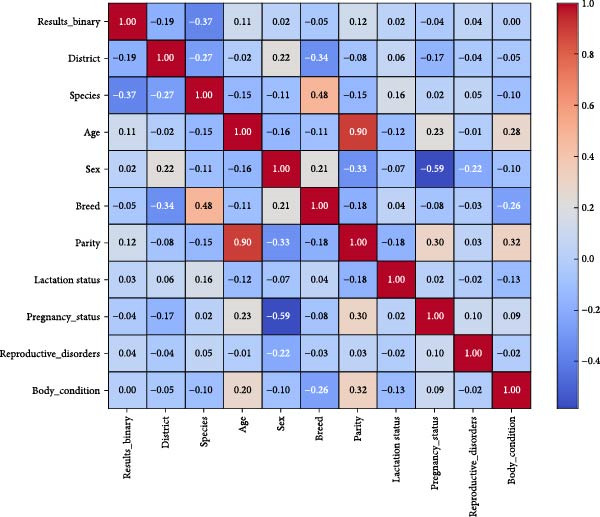
Correlation heatmap of PPRV seropositivity and associated predictors.

Figure 10 shows the Pearson correlation coefficients that link binary ELISA results with demographic, reproductive, and physiological predictors. The numbers shown in each cell are the Pearson correlation coefficients (*r*‐values) that were obtained between pairs of variables. These coefficients represent a normalized scale between −1.0 and +1.0, values close to +1.0 are indicative of a strong positive relationship and values close to −1.0 are indicative of a strong negative relationship. Coefficients that have values near zero show that there is no significant linear relationship. Red, blue and lighter one colors are associated with positive, negative, and weaker or nonexistent association, respectively. Research data indicates a strong positive relationship between parity and age (*r* = 0.90) alongside a positive relationship between species and breed (*r* = 0.48) and negative correlation between sex and pregnancy status (*r* = −0.59). ELISA test results for positivity (Results_binary) exhibit low correlations with other variables meaning that several predictors jointly explain seropositivity better than individual variables do when considered alone.

## 4. Discussion

Research findings show a high seroprevalence of PPRV (79.77%) among unvaccinated sheep and goat populations across various districts of Punjab, Pakistan. The serological data indicate strong viral circulation in the region, consistent with previous research conducted in similar agricultural settings. Comparable studies have also reported high PPRV seroprevalence rates, including 76.7% in Ethiopia [22], 88.2% in Tanzania [23], 81.6% in Sudan [24], and 70.5% in Bangladesh [25]. PPRV transmission is influenced by key ecological and epidemiological factors in a given area, as the district‐specific seroprevalence values ranged from 52.05% in Okara to 100% in Nankana. Research from Nigeria [26] and Nepal [27] demonstrated similar spatial heterogeneity, where flock mixing, seasonal transhumance, and vaccination gaps led to significant differences in local seroprevalence. Study districts with higher goat densities and mixed‐species herding systems, including Nankana and Bhakkar, showed increased seroprevalence levels, due to enhanced interspecies transmission as reported previously [28].

The results of this study showed that goats had a high seroprevalence (90.27%), whereas sheep exhibited a comparatively lower seroprevalence (68.75%). This species difference is in tandem with global studies that report goats are clinically more susceptible to PPRV than sheep [29, 30]. The pathogenesis of PPR in goats includes a higher level of viremia and tissue lesions leading to increased viral shedding and transmission [31]. Additionally, it has been argued that husbandry practices such as frequent movement and marketing of goats compared to sheep enhance their exposure risk [32].

Seroprevalence generally showed an upward trend with age, reaching the highest level in animals aged between 8 and 10 years, despite some variation among intermediate age groups. This implies that seropositivity results from cumulative exposure to these factors over time, as also observed in the previous studies from India [33] and Ethiopia [34]. Interestingly, a seroprevalence of 83.51% was also recorded in young animals aged 6 months to 2 years; this may have resulted from vertical transmission, environmental contamination, or contact with infected older animals in regions with inadequate biosecurity measures [17].

The study also revealed that male animals had a higher prevalence (85.77%) than female animals (76.54%). These findings align with the previous studies conducted in Sudan and Nigeria [24, 35], where herd management tends to keep fewer males for breeding—a practice that enhances the chances of re‐exposure. However, immunosuppression in males due to stress during either transportation or mating periods may further increase their susceptibilities [36].

The breeds may differ in susceptibility, or their husbandry practices may expose them differently, leading to divergent seroprevalence across breeds. The highest seroprevalence was observed in Makhi Cheeni (100%) and Beetal (97.72%) breeds, while the lowest was recorded in Lohi and Sindhi breeds. Previous studies have discussed the hereditary component of PPRV susceptibility [37], although proximity to areas with high viral prevalence also contributes to infection risk [32]. Notably, some breeds, such as Lohi, are raised under sedentary conditions with limited mobility, reducing their exposure to these risk factors. Breeds represent discrete taxonomic subdivisions within a species; however, we modelled breed as a categorical variable and species as a distinct covariate to enable comparison of relative seropositivity odds across all breeds sharing similar exposure contexts. Therefore, the OR between a goat breed and a sheep breed reflects the relative epidemiological risk in mixed flock systems rather than a direct association with biological susceptibility. In Punjab, where sheep and goat breeds often compete for the same grazing and water resources, high seropositivity rates in some goat breeds likely reflect both intrinsic factors (e.g., genetic or immune‐related) and extrinsic factors (e.g., movement patterns and husbandry practices). In contrast, more sedentary sheep breeds like Lohi have experienced lower exposure. These findings support the necessity and rationale for breed‐ or management‐specific mitigation strategies alongside species‐centric control.

Parity was not statistically related to the seropositivity (*p* = 0.5092), though increasing parity showed a trend similar to the age‐ and reproductive stress‐related increase in these risks. Lactating animals had a lower prevalence of PPRV antibodies than nonlactating animals. This difference might be attributed to the immunosuppressive effects of lactation, which could reduce the likelihood of seroconversion, influence behavior, or reflect different housing practices for suckling animals. In light of these findings, pregnancy status did not significantly affect seroprevalence, however, nonpregnant animals showed a relatively higher positivity rate. Similar trends have also been observed in other similar serological studies [25, 26].

No statistically significant associations were observed for reproductive disorders, despite a trend toward higher prevalence of abortions and stillbirths in animals, again indicating the potential reproductive implications of PPRV or coinfections in the context of immunosuppression. Even though PPRV does not directly cause reproductive failure in animals, associated secondary bacterial infections or immune pathology should also be considered [38].

Subsequent multivariate logistic regression analysis and marginal effect plots revealed that species, sex, and the interactions between species and district, as well as between age and sex, were likely to increase the likelihood of PPRV seropositivity. In fact, breed was found to be relevant only when included in combination with other variables, further emphasizing the utility of interaction terms in disease risk models. Such interactions among the aforementioned demographic, spatial, and behavioral factors necessitate comprehensive interventions that target each potential route of the disease spread [5, 39].

This study has several significant strengths but also has a few limitations that should be acknowledged. First, the researchers used a commercially available cELISA. As a common practice, this carries a risk of false negatives and false positives, both of which could affect the estimates of true prevalence, albeit modestly. Second, the percentage values in the subgroup analyses—such as those by breed, age category, or geographic district—were based on relatively small sample sizes. This factor may inflate or distort the results, requiring cautious interpretation. Third, the survey lacked clinical observations, or farmers documented disease historical data that would have enabled direct correlation of seroprevalence with on‐farm effects. However, existing literature from Pakistan and surrounding regions consistently reports frequent clinical PPR outbreaks with high morbidity and mortality in goats, consistent with the very high seroprevalence observed in this study. Future studies incorporating serological monitoring, outbreak reporting, clinical evaluation, and long‐term data collection would provide a clearer understanding of PPRV epidemiology and its true impact on small ruminant populations.

When scrutinizing these records, we should not overlook the possibility that vaccination status could influence seroprevalence. In Punjab Province, although vaccination campaigns are conducted, they lack standardisation. It is estimated that only one‐third of small ruminants in the province are vaccinated, and this uneven coverage perpetuates endemic PPRV transmission. As such, the present study only sampled unvaccinated animals, indicating that any positive sera detected in cELISAs reflected exposure to circulating PPRV rather than vaccine‐mediated immunity. The test employed does not follow the principles of Differentiating Infected from Vaccinated Animals (DIVA) testing; however, excluding vaccinated animals reduced misclassification bias. Nonetheless, in regions with widespread vaccination, interpretation of seroprevalence requires caution, as antibody detection may result from either natural infection or vaccine‐induced immunity. These findings highlight the importance of maintaining more detailed vaccination records, expanding vaccination coverage, and utilizing DIVA‐compatible diagnostic tests in the future to enhance PPR surveillance and eradication efforts.

Due to suboptimal vaccination schedules and a lack of adequate diagnostic networks in rural Pakistan, seroprevalence data like this are useful for defining the risk zones, and determining vaccination schedules. Currently, the WOAH and the Food and Agriculture Organization are implementing a PPR elimination initiative, targeting elimination by 2030 [12]; however its success depends on region‐specific data. Thus, the present study provides valuable epidemiological data from one of the highest‐risk areas in Pakistan.

## 5. Conclusion

The overall very high seroprevalence and strong district‐level heterogeneity show that geographically focused vaccination and monitoring campaigns are needed, particularly in hotspot regions such as Nankana, Bhakkar, and Faisalabad. In addition, given the higher vulnerability of goats compared with sheep and the extreme seropositivity of some breeds (e.g., Makhi Cheeni and Beetal), the importance of species‐ and breed‐specific risk‐profiling in interventions is further emphasized. The significant interaction effects that were found (including species with district and age with sex) also indicate that PPRV transmission is influenced by multifaceted demographic and ecological factors. These findings provide a solid evidence framework to develop risk‐based vaccination strategies, enhanced farmer education, and improved regional surveillance networks, which will assist Pakistan in contributing to the global PPR elimination objective by 2030.

## Ethics Statement

The authors have nothing to report.

## Conflicts of Interest

The authors declare no conflicts of interest.

## Author Contributions

Conceptualization: Mughees Aizaz Alvi, Hong‐Bin Yan, Muhammad Saqib, Li Li, and Yongxi Dou. Methodology: Mughees Aizaz Alvi, Rai Bahadur Kharl, Li Li, Muhammad Saqib, and Yongxi Dou. Investigation: Mughees Aizaz Alvi, Rai Bahadur Kharl, Aliza Ali, Muhammad Zeeshan, and Muhammad Saqib. Formal analysis: Mughees Aizaz Alvi, Rai Bahadur Kharl, and Muzafar Ghafoor. Data curation: Li Li, Wan‐Zhong Jia, Shanhui Ren, Xuelian Meng, Xueliang Zhu, and Yongxi Dou. Writing – original draft: Mughees Aizaz Alvi. Writing – review and editing: Hong‐Bin Yan, Wan‐Zhong Jia, and Yongxi Dou. Supervision: Hong‐Bin Yan, Wan‐Zhong Jia, Muhammad Saqib, and Yongxi Dou. Software: Li Li, Shanhui Ren, Xuelian Meng, and Xueliang Zhu. Project administration: Mughees Aizaz Alvi, Hong‐Bin Yan, Muhammad Saqib, and Yongxi Dou. Mughees Aizaz Alvi, Hong‐Bin Yan and Rai Bahadur Kharl are the co‐first authors and contributed equally to this study.

## Funding

This study was supported by the National Key Research and Development Program (Grant 2022YFD1302100), International Science & Technology Innovation Program of Chinese Academy of Agricultural Sciences (CAASTIP‐2025–05 and CAAS‐ASTIP‐2021‐LVRI), The Major Science and Technology Project of Gansu Province (Grants 24ZD13NA008, 23ZDNA007, and 22ZD6NA001), Central Public‐Interest Scientific Institution Basal Research Fund (Grant 1610312023012), the Fundamental Research Funds for the Central Universities (lzujbky‐2024‐ydyl02), and NBCITS (CARS‐37).

## Supporting Information

Additional supporting information can be found online in the Supporting Information section.

## Supporting information


**Supporting Information S1** PPRV epidemiological data recording form.

## Data Availability

All data associated with this study are included in this manuscript.
