# Supplementary material for: Seroprevalence and Associated Risk Factors of Peste des Petits Ruminants Virus in Small Ruminants of Punjab, Pakistan
Source: Transbound Emerg Dis. 2026 Jan 19;2026:7531764. doi: 10.1155/tbed/7531764 (PMC12815611; doi:10.1155/tbed/7531764)
Supplement: Supplementary file 1 — Supporting Information S1 PPRV epidemiological data recording form. [file TBED-2026-7531764-s001.docx]

**Section A: Owner Information**

1. Owner Name: _______________________________
2. Contact Number: ____________________________
3. Address (Village/Town, District): _______________________________
4. CNIC (optional): ____________________________
5. Farm/Household Size (No. of animals): _______

**Section B: Animal Information**

1. **District:**
   ☐ Bhakkar
   ☐ Faisalabad
   ☐ Layyah
   ☐ Nankana
   ☐ Okara
   ☐ Rajanpur
2. **Species:**
   ☐ Goats
   ☐ Sheep
3. **Sex:**
   ☐ Female
   ☐ Male
4. **Age Group:**
   ☐ 0–2 years
   ☐ 2–4 years
   ☐ 4–6 years
   ☐ 6–8 years
   ☐ 8–10 years
5. **Breed:**
   ☐ Beetal
   ☐ DDP
   ☐ Kajli
   ☐ Lohi
   ☐ Makhi Cheeni
   ☐ Mundri
   ☐ Nachy
   ☐ Rajanpuri
   ☐ Sindhi
   ☐ Teddy
   ☐ Thalli

**Section C: Reproductive Information (For Females Only)**

1. **Parity (Number of times given birth):**
   ☐ 0 (Zero / Heifer)
   ☐ 1st
   ☐ 2nd
   ☐ 3rd
   ☐ 4th
   ☐ 5th
   ☐ 6th
   ☐ 7th
   ☐ 8th
   ☐ 9th
2. **Lactation Status:**
   ☐ Lactating
   ☐ Non-lactating
3. **Pregnancy Status:**
   ☐ Non-pregnant
   ☐ Pregnant
4. **Reproductive Disorders:**
   ☐ Abortion
   ☐ Nil
   ☐ Premature Delivery
   ☐ Repeat Breeder
   ☐ Stillbirth

**Enumerator Name:** ________________________

**Date of Data Collection:** __________________
